# Laparoscopic management of a gastric diverticulum: Case report and literature review

**DOI:** 10.1097/MD.0000000000043858

**Published:** 2025-08-08

**Authors:** Youssef Sleiman, Ribal Aby Hadeer, Hadi Farhat, Razan Moghnieh, Nawaf Jurdi, Leila Abs, Mustafa Alloush

**Affiliations:** a Faculty of Medical Sciences, Lebanese University, Beirut, Lebanon; b Department of General and Visceral Surgery, Nini Hospital, Tripoli, Lebanon; c Department of General Surgery, University of Balamand, Beirut, Lebanon; d Department of Anatomo-Pathology, Nini Hospital, Tripoli, Lebanon; e Department of Radiology, Nini Hospital, Tripoli, Lebanon.

**Keywords:** case report, epigastric pain, gastric diverticulum, laparoscopy, stomach, surgery

## Abstract

**Rationale:**

Gastric diverticulum (GD) is a rare condition characterized by an abnormal bulging pouch in the stomach wall, most commonly located in the fundus. Although GD is typically asymptomatic, it can lead to various nonspecific upper gastrointestinal symptoms, posing a challenge for diagnosis and management.

**Patient concerns:**

A 28-year-old female patient presented with morning emesis, which prompted an investigation and the eventual diagnosis of a symptomatic GD. The patient’s condition was unusual due to her young age and the presence of symptoms typically not associated with GD.

**Diagnoses:**

The diagnosis of GD was confirmed after a series of diagnostic tests that included an abdominal ultrasound, abdominal magnetic resonance imaging and barium swallow. The condition is typically rare in younger individuals and can be easily overlooked due to the nonspecific nature of its symptoms.

**Interventions:**

An esophagogastroduodenoscopy was ordered to exclude other pathology and showed a gastric fundal diverticulum with preserved architecture and no signs of inflammation. The patient was successfully treated using a laparoscopic approach, which is becoming the treatment of choice for large or complicated gastric diverticula.

**Outcomes:**

The laparoscopic surgery resulted in the resolution of the patient’s symptoms, with no reported complications post-surgery. The patient had a favorable recovery and was discharged with no ongoing gastrointestinal issues.

**Lessons:**

This case emphasizes the importance of considering GD in differential diagnoses for patients presenting with vague upper gastrointestinal symptoms, even in younger individuals. It also highlights the effectiveness of laparoscopic surgery as a minimally invasive and successful treatment option for GD.

## 1. Introduction

Gastric diverticula (GD) are defined as a pathological outpouching of the stomach wall. They are classified as either acquired or congenital, and true or false.^[1]^ The most common site is the posterior wall of the gastric fundus, 2 cm below the gastroesophageal junction and 3 cm from the lesser curvature.^[1]^ They are the least common gastrointestinal tract diverticula, sharing similar features to small bowel and colonic diverticula.^[2]^ Their incidence will depend on the procedure done to discover them. Estimates of prevalence range from 0.04% in upper gastrointestinal (UGI) contrast radiographic studies to 0.01% to 0.11% in upper gastrointestinal endoscopies to 0.02% in autopsy studies.^[1,3,4]^ Symptoms of GD vary and can imitate those of other common disorders.^[5,6]^ UGI contrast studies and esophagogastroduodenoscopy (EGD) are considered the most dependable diagnostic tools for confirming the presence of a gastric diverticulum (GD).^[6]^ Although most GD are asymptomatic and managed conservatively with antispasmodics, antacids and dietary modifications, medical therapy such as proton pump inhibitors (PPIs) may be used, particularly when gastroesophageal pathology is present. Prokinetic agents have also shown benefit in promoting emptying of larger diverticula.^[7]^ However, in cases involving large diverticula (>4 cm), persistent symptoms despite medical management, or complications, surgical resection – preferably via a laparoscopic approach – is associated with favorable outcomes, with approximately two-thirds of patients experiencing symptom resolution postoperatively.^[6,8]^ The rarity of this disorder, as well as the presence of vague and nonspecific symptomatology, necessitates a high clinical index of suspicion from the internist, gastroenterologist, or surgeon.

## 2. Case presentation

This is a case of a 28-year-old female patient previously healthy and presented for morning emesis, dyspepsia, and epigastric pain, refractory to PPI treatment. The patient is a smoker (10 pack-year) with a body mass index of 20.7, taking oral contraceptive pills, and has no known food or drug allergies. She denied alcohol or illicit drug use.

On clinical examination, the patient was afebrile with normal vital signs (blood pressure: 112/74 mm Hg; heart rate: 78 bpm). Abdominal examination revealed a soft, non-tender, non-distended abdomen with no guarding or rebound tenderness. Bowel sounds were normal. There was no palpable mass or hepatosplenomegaly.

Initial laboratory investigations, including a complete blood count, liver function tests, pancreatic enzymes (amylase and lipase), renal function panel, electrolytes, C-reactive protein, and erythrocyte sedimentation rate, were all within normal limits (Table 1).

**Table 1:** Initial laboratory investigations.

Test	Patient value	Reference range
Complete blood bount (CBC)		
Hemoglobin	13.4 g/dL	12.0–15.5 g/dL
White blood cells (WBC)	6700/µL	4000–11,000/µL
Platelets	2,53,000/µL	1,50,000–4,00,000/µL
Liver function tests (LFTs)		
AST (SGOT)	22 U/L	10–40 U/L
ALT (SGPT)	18 U/L	7–56 U/L
Alkaline phosphatase	94 U/L	44–147 U/L
Total bilirubin	0.7 mg/dL	0.1–1.2 mg/dL
Pancreatic enzymes		
Amylase	58 U/L	30–110 U/L
Lipase	41 U/L	0–160 U/L
Renal function panel		
BUN	13 mg/dL	7–20 mg/dL
Creatinine	0.9 mg/dL	0.6–1.1 mg/dL
Electrolytes		
Sodium	141 mmol/L	135–145 mmol/L
Potassium	4.1 mmol/L	3.5–5.1 mmol/L
Chloride	102 mmol/L	98–107 mmol/L
Bicarbonate (HCO₃⁻)	24 mmol/L	22–29 mmol/L
Inflammatory markers		
C-reactive protein (CRP)	<1.0 mg/L	<5.0 mg/L
Erythrocyte sedimentation rate (ESR)	10 mm/h	<20 mm/h (female)

ALT = alanine aminotransferase, AST = aspartate aminotransferase, BUN = blood urea nitrogen, CBC = complete blood count, CRP = C-reactive protein, ESR = erythrocyte sedimentation rate, LFTs = liver function tests, SGOT = serum glutamic-oxaloacetic transaminase, SGPT = serum glutamic-pyruvic transaminase, WBC = white blood cells.

The initial differential diagnosis included functional dyspepsia, peptic ulcer disease, gastroesophageal reflux disease, gastritis, and gallbladder pathology. Less common considerations included pancreatic disorders, gastroparesis, and gastrointestinal structural abnormalities such as hiatal hernia or gastric volvulus. Exclusion of possible mass-effect symptoms and anatomical causes were also considered.

**Figure 1.**
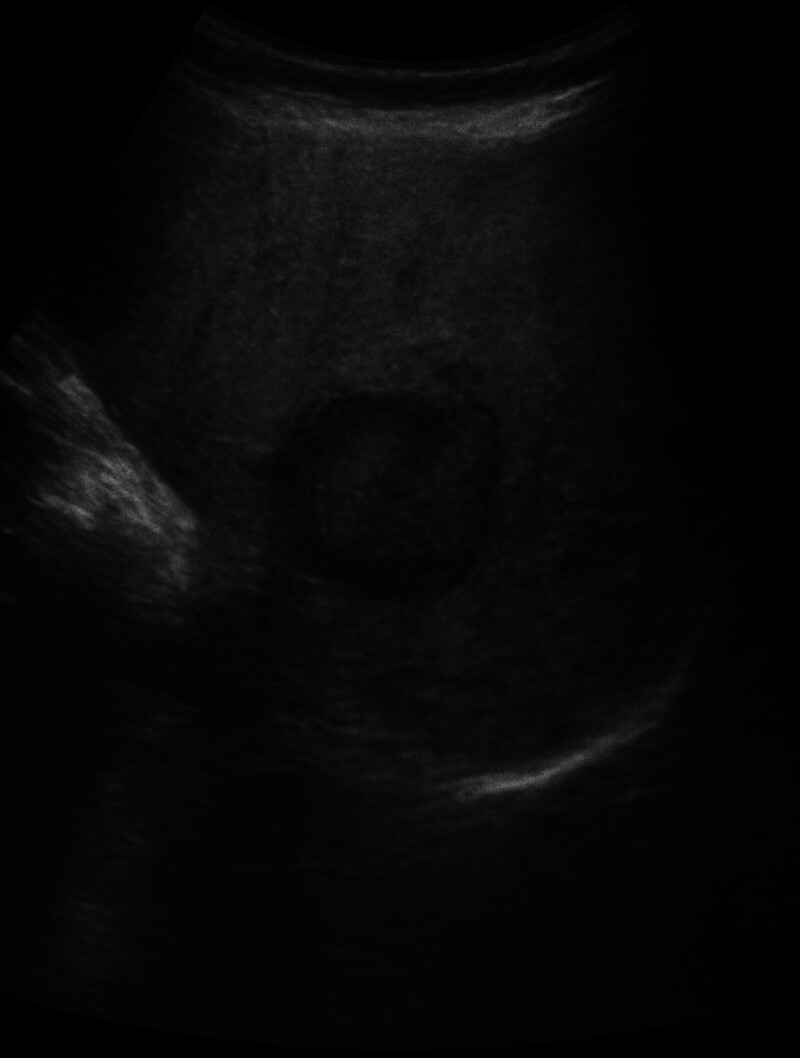
Abdominal ultrasound showing a 2 cm hepatic adenoma.

**Figure 2.**
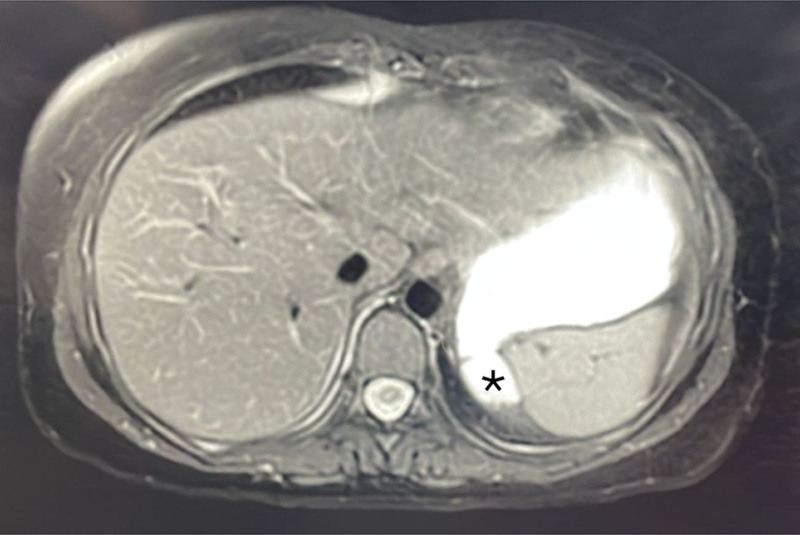
Abdominal MRI: The asterisk designates the gastric diverticulum at the level of the posterior fundus. MRI = magnetic resonance imaging.

Abdominal ultrasound was ordered, it was normal except for a 2 cm hepatic adenoma in Segment VI of the liver (Fig. 1). She did an abdominal magnetic resonance imaging to evaluate this adenoma, it showed a GD of 1.5 cm at the level of the fundus (Fig. 2).

**Figure 3.**
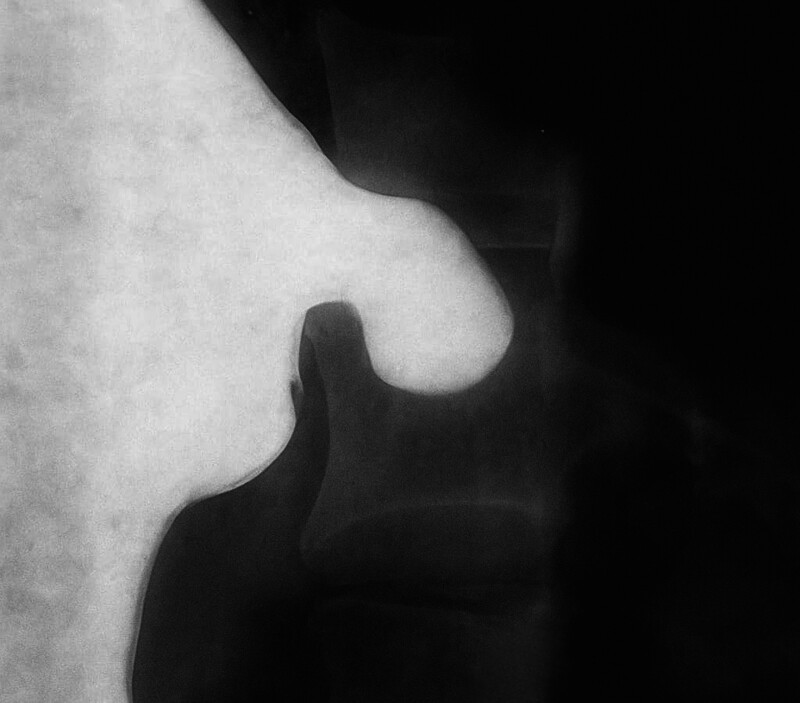
Barium swallow showing the presence of a fundal gastric diverticulum.

Barium swallow confirmed the presence of a GD located in the fundus, in addition to mild gastroesophageal reflux (Fig. 3).

**Figure 4.**
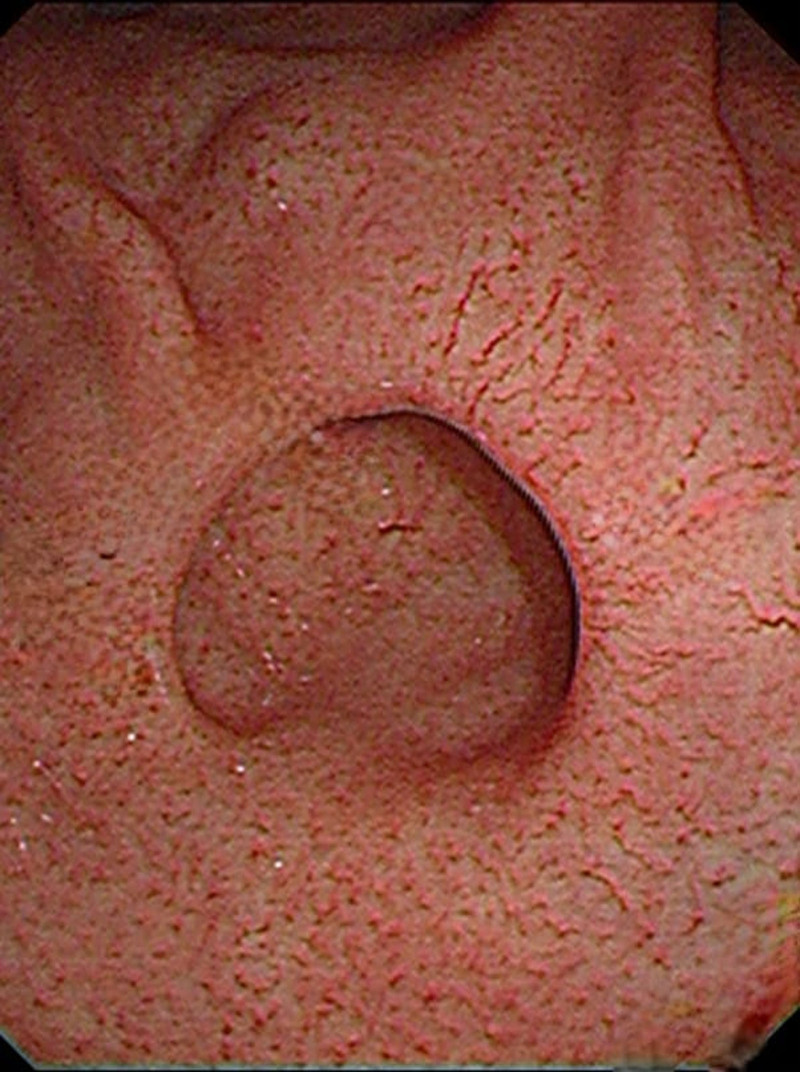
Esophagogastroduodenoscopy presenting the gastric diverticulum at the fundal level posteriorly.

**Figure 5.**
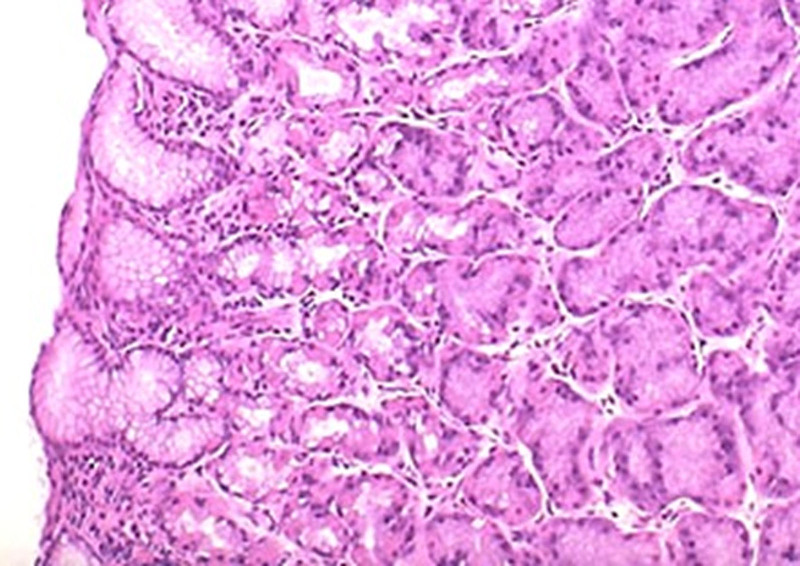
Gastric mucosa demonstrating healthy stomach lining with no signs of disease, inflammation, or infection and a normal cellular composition and organization.

An EGD was ordered to exclude other pathology. A gastric fundal diverticulum with a diameter of 1.5 cm was noted (Fig. 4), and microscopic pathological studies showed preserved architecture and no signs of inflammation in the esophagus, stomach, and duodenum (Fig. 5). Campylobacter-like organism test was negative. The diagnosis was therefore established.

**Figure 6.**
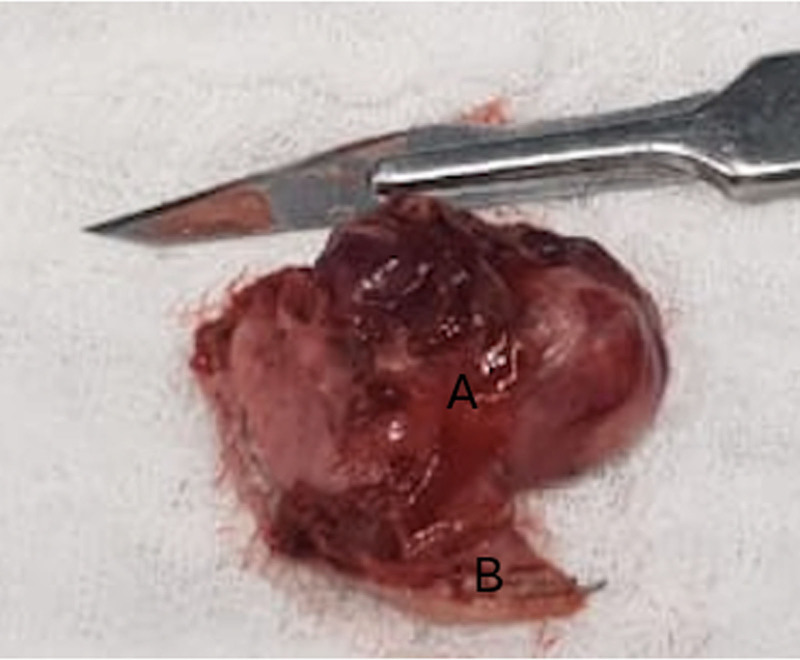
The gastric diverticulum specimen compared to the blade number 11. (A) the body of the GD, (B) the base of the GD. GD = gastric diverticulum.

The patient was then prepared for a laparoscopic intervention, during which the diverticulum, originating from the posterior fundus, was identified. Proximal partial gastrectomy, targeting the origin of the diverticulum, was performed laparoscopically after insertion of a Faucher tube to protect the gastroesophageal junction and aid in anatomical orientation. The patient was positioned in reverse Trendelenburg, and pneumoperitoneum was established. Four laparoscopic ports were inserted. Intraoperative inspection revealed a 1.5 cm GD arising from the posterior aspect of the fundus. The stomach was mobilized by dividing the short gastric vessels using a vessel-sealing device to gain access to the posterior wall. The diverticulum was excised en bloc with the adjacent gastric wall using an endoscopic linear stapler, ensuring clear margins and preservation of the gastroesophageal junction. Hemostasis was confirmed, and the specimen was extracted (Fig. 6). The procedure was completed without complications, and all port sites were closed in layers.

**Figure 7.**
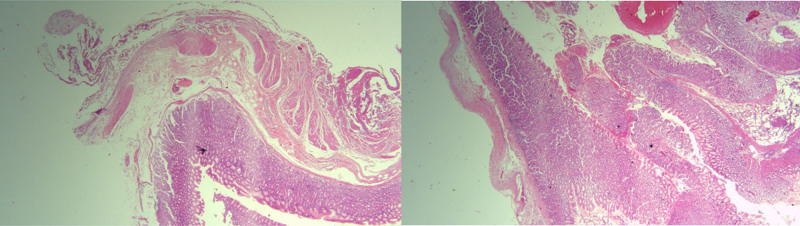
Low power view (2×) of the full thickness gastric wall showing the attenuated muscularis propria layer associated with the diverticulum.

The gastric pouch removed was sent for pathological studies that confirmed the diagnosis of fundal GD (1.5 cm; Fig. 7).

**Figure 8.**
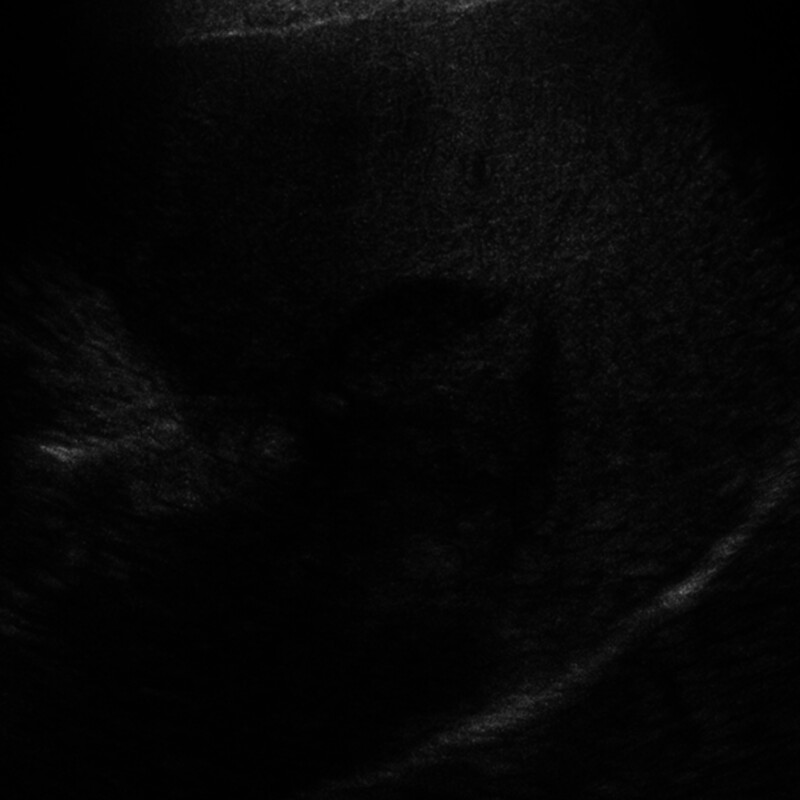
Follow-up abdominal ultrasound showing a stable 2 cm hepatic adenoma.

Given the suspected association between the hepatic adenoma and estrogen-containing oral contraceptive pills, the patient was transitioned to a non-estrogen-containing alternative. The patient was stable postoperatively and was discharged home on postoperative day 2 after an uneventful stay. The patient remained asymptomatic on follow-up at 1 month, 3 months, and 1 year. At 1 month, an abdominal ultrasound showed a stable hepatic adenoma and no postoperative complications (Fig. 8). At 3 months, a barium swallow demonstrated normal gastric contour with no evidence of residual or recurrent diverticulum. At 1 year, an EGD revealed normal mucosa and confirmed the absence of recurrence. Written informed consent was obtained from the patient for the publication of this case report.

## 3. Discussion

GD can either be congenital or acquired, with congenital being more common.^[1,5,9]^ It is hypothesized that congenital diverticula develop due to the dividing of the longitudinal muscle fibers at the level of the cardia, leaving only the circular muscle fibers in the wall of the stomach and, as a result, forming a weak area through which a diverticulum can develop during the fetal period.^[6]^ This theory is supported by reports of GD in the fetus.^[10,11]^ This defect allows mucosal herniation during fetal development, typically occurring along the dorsal fundus near the gastroesophageal junction. Additionally, some cases involve aberrant pancreatic tissue in prepyloric regions or abnormal traction from Grassi’s nerve (a branch of the posterior vagal trunk). These diverticula are classified as “true” diverticula because they contain all layers of the gastric wall, distinguishing them from acquired forms.^[6,7]^

Acquired gastric diverticula in contrast are pseudodiverticula, less common and typically located in the antrum. They usually present with a background history of other gastrointestinal pathologies, such as peptic ulcer disease, malignancy, pancreatitis, or gastric outlet obstruction. Gastric diverticula had been reported following surgical procedures on the stomach, including Roux-en-Y gastric bypass.^[5,12–14]^

This hypothesis suggests therefore that our young patient, with no previous medical or surgical history, had a probably congenital GD of the fundus that remained asymptomatic till shortly before the presentation date.

The distribution of GD is identical between male and female patients, typically appearing in the fifth and sixth decade of life, with only 4% of GD occurring exceptionally in patients younger than 20 years old.^[8]^ The discovery of GD in our 28-year-old patient is thus an atypical and uncommon finding.

GD usually measures 4 cm with a range of 3 to 11 cm and in general, are solitary lesions. Since their small diameter, most GD are asymptomatic and a large percentage of patients will remain asymptomatic throughout life, whether they will be symptomatic, depends on the size, width of the base, location, and the presence of ectopic tissue within the diverticulum. There are no pathognomonic symptoms to suspect GD; patients with symptomatic gastric diverticula will complain of variable nonspecific UGI symptoms including a sensation of fullness in the upper abdomen, less commonly dyspepsia, vomiting, intermittent regurgitation of solid foods, belching, halitosis, and dysphagia, making it a diagnostic challenge for physicians necessitating a high clinical index of suspicion.^[5,15]^ Additional rare reported complications such as bleeding, perforation, ulceration, and malignant transformation can develop requiring urgent surgical intervention. In our case, the patient showed symptoms (dyspepsia, morning vomiting, and epigastric pain) compatible with gastroesophageal reflux disease, hence she was treated by PPI before presentation to our clinic. Imaging (barium swallow and magnetic resonance imaging) were then responsible for evoking the differential diagnosis of GD.

Most GD do not require treatment. Conservative therapy consisted primarily of antispasmodics, antacids, preprandial paraffin, and/or a soft diet with postural drainage. PPIs can be used to treat the commonly associated gastroesophageal pathology, and prokinetic agents have been used to encourage the emptying of larger GD.^[7]^

For large GD (>4 cm in diameter), persistent symptoms despite PPI treatment, and complicated diverticula, surgical resection whether open or laparoscopic will lead to excellent results with two-thirds of patients remaining symptom-free after the surgery,^[7]^ although, currently, the laparoscopic approach is preferred.^[6]^

In our case, the patient’s symptoms didn’t seem to ameliorate despite treatment with PPI, which led us to opt for laparoscopic partial gastrectomy to remove the diverticulum. This has led to the patient’s recovery with no symptoms.

It’s worth noting that, despite the uncommonly small diameter of the diverticulum (1.5 cm), the GD was symptomatic, resistant to medical treatment, and required surgical intervention.

The work has been reported in line with the CARE criteria.^[16]^

## 4. Conclusion

GD is a rare disease with a nonspecific presentation; therefore, the diagnosis can be tricky. Physicians and surgeons should always think of ruling out GD when dealing with a patient having general gastrointestinal or abdominal symptoms with no apparent cause. The choice of treatment should be made following the severity of symptoms and the size of the diverticulum, and it should not be delayed to avoid rare but rather fatal complications of the disease. Meanwhile, laparoscopic surgery is the treatment of choice in case of large and complicated gastric diverticula.

## Author contributions

**Conceptualization:** Youssef Sleiman, Ribal Aby Hadeer, Razan Moghnieh.

**Data curation:** Youssef Sleiman.

**Project administration:** Razan Moghnieh.

**Supervision:** Youssef Sleiman, Razan Moghnieh, Leila Abs, Mustafa Alloush.

**Validation:** Razan Moghnieh.

**Writing – original draft:** Ribal Aby Hadeer, Hadi Farhat, Nawaf Jurdi.

**Writing – review & editing:** Ribal Aby Hadeer, Hadi Farhat, Razan Moghnieh, Nawaf Jurdi, Leila Abs, Mustafa Alloush.

## References

[1] Rodeberg DA Zaheer S Moir CR Ishitani MB. Gastric diverticulum: a series of four pediatric patients. J Pediatr Gastroenterol Nutr. 2002;34:564–7. 12050587 10.1097/00005176-200205000-00019

[2] Chen JH Su WC Chang CY Lin H. Education and imaging. Gastrointestinal: bleeding gastric diverticulum. J Gastroenterol Hepatol. 2008;23:336. 18289361 10.1111/j.1440-1746.2007.05301.x

[3] Gockel I Thomschke D Lorenz D. Gastrointestinal: gastric diverticula. J Gastroenterol Hepatol. 2004;19:227–227. 14731136 10.1111/j.1440-1746.2004.3339b.x

[4] Donkervoort SC Baak LC Blaauwgeers JL Gerhards MF. Laparoscopic resection of a symptomatic gastric diverticulum: a minimally invasive solution. JSLS. 2006;10:525–7. 17575774 PMC3015748

[5] Rashid F Aber A Iftikhar SY. A review on gastric diverticulum. World J Emerg Surg. 2012;7:1. 22257431 10.1186/1749-7922-7-1 PMC3287132

[6] Shah J Patel K Sunkara T Papafragkakis C Shahidullah A. Gastric diverticulum: a comprehensive review. Inflamm Intest Dis. 2019;3:161–6. 31111031 10.1159/000495463 PMC6501548

[7] Morris PD Allaway MGR Mwagiru DK Sinclair JB Hollands M. Gastric diverticulum: a contemporary review and update in management. ANZ J Surg. 2023;93:2828–32. 37743578 10.1111/ans.18707

[8] Palmer ED. Gastric diverticula. Int Abstr Surg. 1951;92:417–28. 14840911

[9] Love L Meyers MA Churchill RJ Reynes CJ Moncada R Gibson D. Computed tomography of extraperitoneal spaces. AJR Am J Roentgenol. 1981;136:781–9. 6784475 10.2214/ajr.136.4.781

[10] Reich NE. Gastric diverticula. Am J Dig Dis. 1941;8:70–6.

[11] Lewis FT Thyng FW. Regular occurrence of intestinal diverticula in embryos of pig, rabbit and man. Am J Anat. 1908;7:505–19.

[12] Meeroff M Gollán JR Meeroff JC. Gastric diverticulum. Am J Gastroenterol. 1967;47:189–203. 4960419

[13] Anaise D Brand DL Smith NL Soroff HS. Pitfalls in the diagnosis and treatment of a symptomatic gastric diverticulum. Gastrointest Endosc. 1984;30:28–30. 6423437 10.1016/s0016-5107(84)72291-7

[14] Schweiger F Noonan JS. An unusual case of gastric diverticulosis. Am J Gastroenterol. 1991;86:1817–9. 1962629

[15] DuBois B Powell B Voeller G. Gastric diverticulum: “a wayside house of ill fame” with a laparoscopic solution. JSLS. 2012;16:473–7. 23318077 10.4293/108680812X13462882736330 PMC3535786

[16] Gagnier JJ Kienle G Altman DG Moher D Sox H Riley D; CARE Group. The CARE guidelines: consensus-based clinical case reporting guideline development. BMJ Case Rep. 2013;2013:bcr2013201554. 10.1186/1752-1947-7-223 PMC3844611 24228906

